# Larger corpus callosum volume is favorable for theory of mind development in healthy children

**DOI:** 10.1093/cercor/bhad353

**Published:** 2023-10-11

**Authors:** Sophie Mandl, Patric Kienast, Kathrin Kollndorfer, Gregor Kasprian, Michael Weber, Rainer Seidl, Lisa Bartha-Doering

**Affiliations:** Department of Pediatrics and Adolescent Medicine, Comprehensive Center for Pediatrics, Medical University of Vienna, Vienna 1090, Austria; Department of Biomedical Imaging and Image-guided Therapy, Medical University of Vienna, Vienna 1090, Austria; Department of Pediatrics and Adolescent Medicine, Comprehensive Center for Pediatrics, Medical University of Vienna, Vienna 1090, Austria; Department of Biomedical Imaging and Image-guided Therapy, Medical University of Vienna, Vienna 1090, Austria; Department of Biomedical Imaging and Image-guided Therapy, Medical University of Vienna, Vienna 1090, Austria; Department of Biomedical Imaging and Image-guided Therapy, Medical University of Vienna, Vienna 1090, Austria; Department of Pediatrics and Adolescent Medicine, Comprehensive Center for Pediatrics, Medical University of Vienna, Vienna 1090, Austria; Department of Pediatrics and Adolescent Medicine, Comprehensive Center for Pediatrics, Medical University of Vienna, Vienna 1090, Austria

**Keywords:** brain structure, MRI, neuroimaging, social cognition, typical development

## Abstract

While previous research has demonstrated a link between the corpus callosum (CC) and theory of mind (ToM) abilities in individuals with corpus callosum agenesis (ACC), the relationship between CC volume and ToM remains unclear in healthy children. The present study examined whether CC volume influences children’s performance on ToM tasks that assess their understanding of pretense, emotion recognition, and false beliefs. Forty children aged 6–12 years underwent structural magnetic resonance imaging (MRI) and a cognitive test battery. We found that larger mid-anterior and central subsections of the CC significantly correlated with better ToM abilities. We could also demonstrate age- and sex-related effects, as the CC–ToM relationship differed between younger (6–8 years) and older (9–12 years) children, and between female and male participants. Importantly, the older children drove the association between the CC mid-anterior and central subsection volumes and ToM abilities. This study is the first to demonstrate that CC size is associated with ToM abilities in healthy children, underlining the idea that the CC plays a vital role in their socio-cognitive development. CC subsection volumes may thus not only serve as a measure of heterogeneity in neurodevelopmental populations known to exhibit socio-cognitive deficits, but also in typically developing children.

## Introduction

The corpus callosum (CC) is the largest interhemispheric white matter tract, consisting of more than 200 million commissural fibers connecting the two cerebral hemispheres (Luders et al. 2010). Anatomically, the CC can be subdivided into four regions, namely the rostrum, genu, body, and splenium (Goldstein et al. 2023). These regions vary depending on the cortical destination and fiber composition (Banich 1995). Small diameter fibers are found in the rostrum, genu, and anterior mid-body, which connect the prefrontal and premotor cortices, and in parts of the splenium, which connect the temporal and parietal cortices (Hofer and Frahm 2006; Park et al. 2008). These cortical areas are known to be involved in higher-order cognitive functions, such as language (Bartha-Doering et al. 2018; Emch et al. 2019). Large diameter fibers, on the other hand, are found in the posterior mid-body and in the posterior portion of the splenium, which transfer motor, somatosensory, auditory, and visual information. It is generally assumed that regional CC size is related to fiber density, especially in areas primarily composed of small diameter fibers (Aboitiz et al. 1992). In turn, previous studies in healthy children and normal-aging adults have demonstrated that a larger CC size relates to an increased capacity for interhemispheric transfer for specific cortical functions, such as verbal fluency (Bartha-Doering et al. 2021b), problem solving (Zahr et al. 2009), and executive function (Voineskos et al. 2012).

It has also been postulated that CC size plays a role in children’s theory of mind (ToM) development, which is the ability to attribute thoughts, feelings, beliefs, and intentions to others and to use this ability to predict their behavior (Mitchell 1997). This ability develops in the preschool years and is thought to be a prerequisite for understanding one’s social environment (Poulin-Dubois 2020). So far, studies exploring the CC–ToM relationship have focused on children and adults with agenesis of the corpus callosum (ACC; Badaruddin et al. 2007; Symington et al. 2010; Turk et al. 2010). These studies have found that, despite an average intelligent quotient (IQ) and normal cognitive functioning (Moutard et al. 2003), individuals with ACC often show deficits in complex social skills that depend on fast interhemispheric processing, such as perspective-taking (Symington et al. 2010; Turk et al. 2010), interpersonal interactions (Badaruddin et al. 2007), and emotion recognition (Bridgman et al. 2014). Deficits are further evident in second-order meanings of language, such as vocal prosody and humor comprehension (Paul et al. 2003).

Previous studies have also revealed smaller CC volumes in autism spectrum disorder (ASD), which is another neurodevelopmental disorder characterized by social impairments, including ToM (Paul et al. 2014). These studies have found significant reductions in the overall CC and in subsections of the CC to be associated with social deficits and cortical underconnectivity (Just et al. 2007; Hardan et al. 2009; Keary et al. 2009; Frazier et al. 2012). It has thus been hypothesized that the social deficits seen in ASD can be traced back to a disruption in interregional connectivity, which relies on the integrity of white matter tracts, such as the CC (Just et al. 2004; Just et al. 2007).

However, given the complexity of these neurodevelopmental disorders, the specific role that the CC plays in the typical development of socio-cognitive functions remains unclear. Adult neuroimaging studies have shown that ToM tasks reliably activate a frontal-posterior network of brain regions, including the medial prefrontal cortex (MPFC), the temporoparietal junction (TPJ), and the temporal poles (Amodio and Frith 2006; Mahy et al. 2014). Yet there is no consensus as to which subsections of the CC may be involved, as reductions have been found in posterior (Just et al. 2007; Freitag et al. 2009), central (Kilian et al. 2007), and anterior regions of the CC in individuals with ASD (Just et al. 2007; Hardan et al. 2009; Keary et al. 2009). Moreover, even though the general architecture of the ToM network is already present in children aged 5–13 years (Kobayashi et al. 2007a; Saxe et al. 2009; Gweon et al. 2012), the neural ToM mechanisms are not functionally mature yet. It is thus unclear to what extent CC subsection volumes could serve as a measure of heterogeneity in ToM abilities in typically developing children as well. The present study therefore aimed to investigate the relationship between CC subsection volumes and ToM abilities in healthy, school-aged children using structural magnetic resonance imaging (MRI) and a cognitive test battery.

## Materials and methods

The present study used methods similar to those in our prior publications (Bartha-Doering et al. 2019, 2023; Mandl et al. 2023). Consequently, some of the text included here is recycled from those sources.

### Participants

Forty healthy children (18 female, 22 male; *M*_age_ = 8.96 years, *SD* = 1.86) were recruited from the community. Inclusion criteria were as follows: (i) no history of neurologic disease; (ii) no clinical evidence of neurologic dysfunction or developmental delay; (iii) aged between 6 and 12 years; (iv) normal or corrected-to-normal vision and hearing; and (v) native German speaking. We chose this age range because it marks a distinctive period between major developmental transition points (National Research Council 1984). All children were enrolled in regular school classes and no child was on medication at the time of the study. The study was approved by the Ethics Committee of the Medical University of Vienna and performed in accordance with the Helsinki Declaration of 1975. All children and one parent or legal guardian per child gave written informed consent before inclusion.

### Background information

The children’s socioeconomic status was evaluated by rating the parents’ educational levels and household income. Educational level was rated separately for each of the two parents and included (1) secondary school, (2) apprenticeship, (3) vocational school, (4) school leaving examination, and (5) university degree. Household income was divided into six ordinal categories: (1) less than 10,000€, (2) 10,000–19,000€, (3) 20,000–29,000€, (4) 30,000–39,000€, (5) 40,000–49,000€, and (6) 50,000€ or more. The socioeconomic status was calculated by taking the arithmetic mean of maternal education, paternal education, and household income as described in Bartha-Doering et al. (2021a).

For background information on general cognitive abilities, we administered two verbal and one nonverbal task. The Token Test for Children was used to measure language comprehension (McGhee et al. 2007). The Wortschatz- und Wortfindungstest was included to assess expressive vocabulary in different lexical categories, including nouns, verbs, adverbs, and adjectives (Glück 2011). The Perceptual Reasoning Index of the Wechsler Intelligence Scale for Children-IV was employed to measure nonverbal IQ (Petermann and Petermann 2010).

### MRI data acquisition

The first 29 of the participants were scanned on a 3T Siemens TIM Trio (Siemens Medical Solutions, Erlangen, Germany). 3D structural MRI scans were performed based on isovoxel magnetization-prepared rapid gradient-echo (MPRAGE, T1-weighted, TE/TR _ 4.21/2,300 ms, inversion time 900 ms, with a matrix size of 240 × 256 × 160, voxel size 1 × 1 × 1.10 mm^3^, flip angle 9°) sequence. Eleven out of 40 children were scanned on a Philips Elition 3T scanner (0.75 × 0.75 × 1 mm^3^, flip angle 8°, TE 3.75 ms, TR 8.188 ms). The MRI scan was always performed within a week of the cognitive test battery.

### C‌C volume calculation

**Fig. 1:**
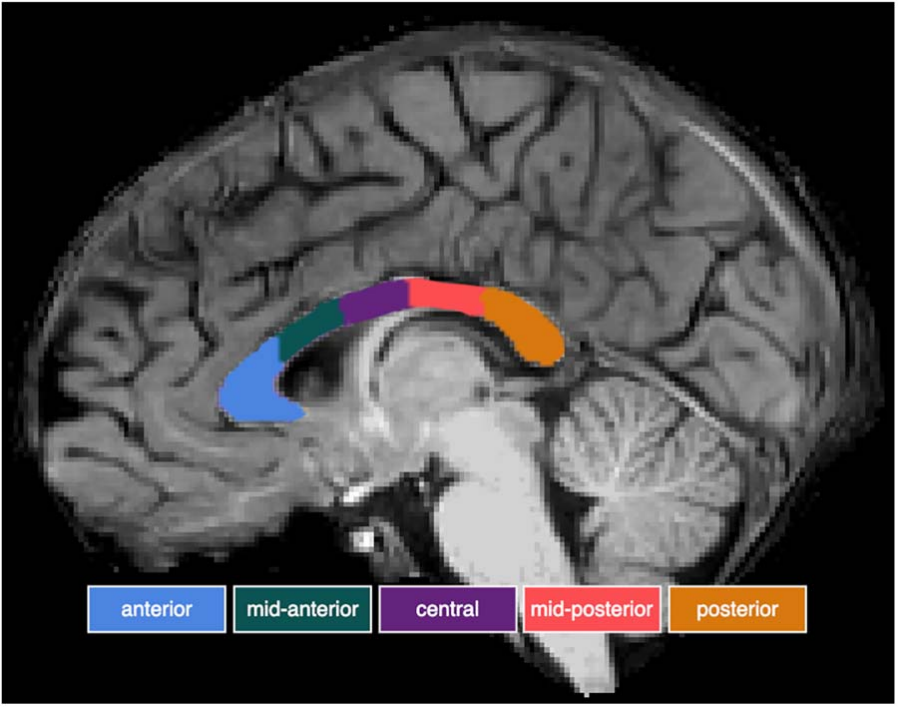
Segmentation of the CC into five subsections using FreeSurfer. The figure design was inspired by Goldman et al. (2017).

The structural MRI images were processed using FreeSurfer version 7.3.2 (https://surfer.nmr.mgh.harvard.edu/). CC subsections were automatically identified and segmented into anterior, mid-anterior, central, mid-posterior, and posterior subsections (Fig. 1; Fischl et al. 2002). Subsection volumes were then computed in FreeSurfer and summed for total CC volume. To control for individual variations in brain size, total and subsection CC volumes were normalized by dividing each volume by the total intracranial volume obtained from FreeSurfer.

### Evaluation of ToM abilities

Children’s ToM abilities were assessed with the *ToM test* developed by Muris et al. (1999). The ToM test is a reliable and valid measure that has been shown to possess good internal consistency (Cronbach’s alpha = 0.92), construct validity, and concurrent validity (see Muris et al. 1999 for more detail). During this task, participants are asked questions about a series of cartoons and stories that evaluate both basic and more advanced aspects of ToM. The test consists of 38 items and 3 subscales: ToM 1 (20 items), ToM 2 (13 items), and ToM 3 (5 items). ToM 1 measures precursors of ToM, including pretense (e.g. “Do as if you comb your hair”), reality versus non-reality (e.g. “Is John able to touch the bike that he dreams about?”), and recognition of others’ emotions (e.g. “How does the boy in the picture feel?”). ToM 2 examines children’s understanding of beliefs and first-order false belief (e.g. “Can you see from the girl’s face how she really feels?”). ToM 3 assesses advanced aspects of ToM, such as second-order false belief and understanding of humor (e.g. “Why does the man say: ‘Wow, we have nice weather today!’”). The child’s answers are then evaluated using the scoring sheet provided in the test booklet, resulting in one score for each subscale and one ToM total score.

### Analysis of cognitive tests

Raw cognitive test scores were transformed into age-corrected *z*-scores. For the ToM test, normative data were only available for the ToM total score. For more detailed analyses of the TOM subscores, we therefore used the raw scores of the three subscales (ToM 1, ToM 2, ToM 3) and controlled for the participants’ age in the partial correlation analyses.

#### Statistical analyses

We conducted all further statistical analyses using SPSS Statistics (version 28.0). As the residuals followed a normal distribution, parametric tests were used. To examine whether ToM scores or CC subsection volumes correlated with the participants’ age or socioeconomic status, Pearson’s correlation analyses were conducted. Mann–Whitney *U* tests were performed to evaluate if the cognitive test scores, CC subsection volumes, socioeconomic status, or age of the participants differed by sex. Pearson’s correlation analyses were then used to measure the association between CC subsection volumes and ToM total *z*-scores in the whole group, age-based groups (6–8 years; 9–12 years), and sex-based groups (female; male). Finally, partial correlation analyses were conducted to measure the association between CC subsection volumes and ToM subscores while controlling for the participants’ age. Significance of correlations was not corrected for multiple comparisons, since CC volumes highly correlated with one another, as did the ToM subscores.

## Results

Outliers in CC and ToM data with more than 3.5 standard deviations from the mean (*n* = 3) were removed from the dataset, resulting in a final sample of 37 participants (18 female, 19 male; Table 1). Given a sample size of 37 cases, the population correlation has to be rho = 0.45 to obtain a power of 80%.

**Table 1:** Study participants (*n* = 37).

	*M* (*SD*)	Range	Age *r* (*p*)	Sex *U* (*p*)
Age	9.00 (1.79)	6.33–12.67	-	135.5 (.281)
Socioeconomic status	3.64 (1.04)	1–5	0.05 (.754)	138.0 (.835)
Language comprehension (*z*-score)	0.66 (0.84)	−1.08—1.88	0.25 (.129)	161.5 (.772)
Vocabulary (*z*-score)	0.41 (1.44)	−3.50—2.05	0.11 (.538)	162.5 (.796)
Nonverbal perceptual reasoning (*z*-score)	0.24 (1.00)	−2.46—1.67	0.07 (.665)	114.5 (.085)

Cognitive background testing revealed that all children had normal language comprehension, with only one child performing slightly below average on the Token Test. Nineteen children demonstrated average, nine children showed above average, six children revealed below average, and three children exhibited impaired expressive vocabulary. Nonverbal perceptual reasoning was average in 25 children and above average in eight children, whereas three children fell below average, and one child showed reduced nonverbal abilities (see Table S1 for more details). In sum, all children could follow the test instructions, and cognitive background testing points to a wide distribution of cognitive performances in the study group.

### C‌C subsection volumes

CC subsection volumes significantly correlated with each other (all *r* ≥ 0.44, *p* ≤ 0.007), except the CC central and CC posterior volumes (*r* = 0.29, *p* = 0.080). Table 2 depicts the results of the volumetric analyses. CC volumes did not correlate with the participants’ age or socioeconomic status. There were also no significant differences in CC subsection or CC total volume by sex. Therefore, we did not control for age, sex or socioeconomic status in subsequent analyses involving CC volumes.

**Table 2:** ToM scores and CC volumes, correlations with age and socioeconomic status, differences by sex.

	*M* (*SD*)	Range	Age*r* (*p*)	Sex*U* (*p*)	SES*r* (*p*)
ToM total (*z*-score)	−0.33 (1.46)	−3.12– 2.04	−0.05 (.752)	157.5 (.682)	0.13 (.482)
ToM 1 (raw score)	24.71 (2.71)	16.80–28.00	−0.01 (.951)	167.0 (.901)	0.18 (.303)
ToM 2 (raw score)	25.00 (4.53)	12.50–32.50	0.43 (.008)*	165.5 (.864)	0.06 (.750)
ToM 3 (raw score)	9.01 (5.54)	0.00–16.50	0.55 (.001)*	165.0 (.852)	0.07 (.681)
CC total (% of IV)	0.22 (.03)	0.17–0.28	0.22 (.187)	158.0 (.693)	0.07 (.697)
CC anterior (% of IV)	0.06 (.01)	0.04–0.08	0.15 (.366)	128.0 (.191)	−0.14 (.441)
CC mid-anterior (% of IV)	0.03 (.01)	0.02–0.04	−0.05 (.778)	150.0 (.523)	0.21 (.237)
CC central (% of IV)	0.04 (.01)	0.02–0.06	0.18 (.292)	164.0 (.832)	0.21 (.233)
CC mid-posterior (% of IV)	0.03 (.01)	0.02–0.04	0.15 (.364)	159.0 (.715)	0.19 (.280)
CC posterior (% of IV)	0.06 (.01)	0.04–0.08	0.26 (.119)	162.0 (.784)	−0.11 (.540)

Note: CC = corpus callosum, SES = socioeconomic status, IV = intracranial volume. Statistical significance is indicated in bold and with an asterisk (*).

### Theory of mind

ToM total *z*-scores revealed average abilities in 14 children, below average abilities in 10 children, and above average abilities in 8 children. Five children showed impaired ToM abilities. The skewness of the ToM total *z*-scores was found to be −.26, indicating a slightly left-skewed distribution. The kurtosis of the ToM total *z*-scores was found to be −.97, suggesting a platykurtic, or light-tailed, distribution. ToM total *z*-scores did not significantly correlate with age or socioeconomic status, nor did the children’s ToM performance differ by sex (Table 2).

With regard to the subscales, participants scored highly on ToM 1, with an average raw score of 24.71 out of 28 points, indicating a ceiling effect. In turn, there was no significant correlation between ToM 1 raw scores and participants’ age (*r* = −.01, *p* = 0.951) nor with the other two subscales (*r* ≤ 0.25, *p* ≥ 0.14). The average raw score on the ToM 2 subscale was 25.00 out of 32.50 points and the average raw score on the ToM 3 subscale was 9.01 out of 16.5 points. Moreover, ToM 2 and 3 raw scores significantly correlated with each other (*r* = 0.59, *p* < 0.001) and with the children’s age (Table 2). Subsequent analyses involving the TOM subscale raw scores were therefore controlled for age.

### Relationship between CC subsection volumes and ToM results

**Fig. 2:**
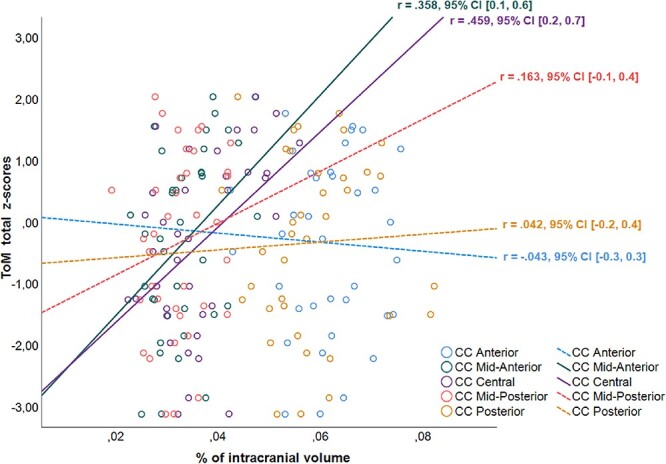
Correlations between CC subsection volumes and ToM total *z*-scores in the whole group. Solid lines indicate significant correlations, while dotted lines indicate non-significant correlations.

In the whole group of participants, we found significant correlations among CC subsection volumes and ToM total *z*-scores. More specifically, ToM total *z*-scores were positively associated with the mid-anterior (*r* = 0.36, *p* = 0.030, 95% CI [0.1, 0.6]) and the central (*r* = 0.46, *p* = 0.004, 95% CI [0.2, 0.7]) subsection volumes of the CC (Fig. 2). Next, we divided the group into two equal age groups to examine whether there are age-related differences in the association between CC volumes and ToM abilities. Even though neither CC volumes nor ToM total *z*-scores significantly correlated with age, the relationship among these variables differed between the two age groups. In the younger group (*n* = 19; aged 6–8 years), there were no significant associations between CC volume and ToM abilities. In the older group (*n* = 18; aged 9–12 years), however, ToM total z -scores were significantly associated with CC mid-anterior (*r* = 0.61, *p* = 0.007, 95% CI [0.2, 0.9]) and central volumes (*r* = 0.58, *p* = 0.012, 95% CI [0.1, 0.9]). In the final step, we tested for sex-related differences by examining the female (*n* = 18) and male (*n* = 19) participants separately. Again, despite our result that CC volumes and ToM total *z*-scores do not differ by sex, we found sex-related differences in the association between these variables. In girls, there was a significant positive correlation between the mid-anterior region of the CC and total ToM *z*-scores (*r* = 0.57, *p* = 0.013, 95% CI [0.2, 0.8]) whereas in boys, the central region of the CC was positively associated with their overall ToM abilities (*r* = 0.55, *p* = 0.014, 95% CI [0.2, 0.8]).

To examine the relationship between CC volumes and the three ToM subscales, we performed a partial correlation analysis, controlling for age. In the whole group analysis, we found a significant association between the CC central volume and ToM 1 (*r* = 0.34, *p* = 0.043, 95% CI [0.0, 0.6]) and ToM 3 scores (*r* = 0.36, *p* = 0.030, 95% CI [0.1, 0.6]). In the younger age group, there were no significant correlations between CC volumes and ToM subscores. In the older age group, ToM 2 correlated with the CC mid-anterior (*r* = 0.63, *p* = 0.007, 95% CI [0.4, 0.8]) and the total volume (*r* = 0.51, *p* = 0.038, 95% CI [0.2, 0.8]), whereas ToM 3 correlated with the CC mid-anterior (*r* = 0.63, *p* = 0.007, 95% CI [0.2, 0.9]) and central volume (*r* = 0.52, *p* = 0.033, 95% CI [0.1, 0.8]). Regarding sex-related differences, we found a negative correlation between the CC anterior volume and the ToM 1 subscale in boys only (*r* = −.50, *p* = 0.034, 95% CI [−0.8, −0.0]).

In sum, the volumes of the CC mid-anterior and central subsections were significantly and positively associated with ToM sub- and total scores in the present sample of healthy, school-aged children. It is important to note that neither language comprehension, expressive vocabulary, nor perceptual reasoning correlated with any of the CC volumes (Table S2). For detailed information on the correlation analyses, please refer to Tables S3 and S4 in the Supplement.

## Discussion

This is the first study to demonstrate a significant relationship between CC subsection volumes and ToM abilities in healthy, school-aged children. When examining the whole group, we found that larger mid-anterior and central subsections of the CC significantly correlated with better overall ToM abilities. Moreover, the CC central subsection was positively associated with specific aspects of ToM, including the ability to understand pretense, differentiate between reality and non-reality, and recognize others’ emotions, and with the ability to understand second-order false beliefs and humor. In the 9- to 12-year-old children, larger mid-anterior, central, and total CC volumes were significantly associated with better scores on first- and second-order false belief tasks and overall ToM abilities. The linkage between the CC subsection volumes and overall ToM abilities also differed between female and male participants, with the mid-anterior subsection volume positively correlating with overall ToM in girls and the central subsection volume positively correlating with overall ToM in boys. Finally, the anterior subsection volume of the CC was negatively associated with precursors of ToM in boys only.

### The role of the CC in interhemispheric processing

Our finding that larger CC volume correlates with better overall ToM scores supports the notion that the CC plays an excitatory role in interhemispheric processing (Bloom and Hynd 2005). According to the theory of excitation, the CC integrates information from both hemispheres, increasing the amount of cortex that can be dedicated to any given task (Yazgan et al. 1995). This theory stands in contrast to the theory of inhibition, which posits that the CC provides a pathway through which one hemisphere can inhibit the other, allowing for independent functioning and more efficient interhemispheric processing (Cook 1984). While there is evidence to support both theories, the excitatory function may be particularly important for tasks that are highly demanding and entail multiple steps in processing (Banich 1995). ToM is an example of a challenging, multi-step task, as it requires the individual to first decode others’ mental states based on observable information and then reason about those mental states to explain or predict others’ actions (Sabbagh 2004). This is in concordance with previous studies on ACC, which consistently report isolated ACC patients to show deficits in social cognition, including ToM (Badaruddin et al. 2007; Symington et al. 2010; Turk et al. 2010). The current study demonstrates that the relationship between CC size and ToM abilities also holds true in healthy children, adding to the growing body of evidence that socio-cognitive functioning relies on interhemispheric pathways connecting the involved brain regions.

The present findings also support previous studies on the role of the CC in ASD (Just et al. 2007; Hardan et al. 2009; Keary et al. 2009; Frazier et al. 2012). These studies have postulated that the social cognition deficits seen in individuals with ASD are related to abnormal CC development and that ACC is associated with a higher risk of developing ASD (Paul et al. 2014). This hypothesis fits with the view that ASD results from a disruption in long-range brain connectivity between higher-order association areas and the frontal lobe (Geschwind and Levitt 2007; Just et al. 2012). It is also consistent with the idea that individuals with ASD have deficits in integrating information that is spatially distributed (Just et al. 2004; Just et al. 2007). With regard to ACC, this would specifically affect abilities that require the integration of cues processed in contralateral hemispheres, such as the comprehension of sarcasm through the combination of verbal output (left hemisphere) and emotional prosody (right hemisphere). If this is the case, then it may be hypothesized that the social impairments seen in both ACC and ASD are due to disruptions in connectivity between regions that need to coordinate with each other to support complex social skills, such as ToM (Kana et al. 2009; Symington et al. 2010; Just et al. 2012; Paul et al. 2014). Given that interregional communication relies on white matter tracts like the CC, reductions in CC size may point to cortical underconnectivity among brain areas involved in ToM processing and thus to deficits in mental state reasoning (Just et al. 2007).

### C‌C subsection volumes associated with ToM abilities

More specifically, we found the mid-anterior and central subsection volumes to be associated with children’s ToM scores. Neuroimaging studies have repeatedly shown that cortical activity is distributed across numerous brain regions during ToM tasks, suggesting that different aspects of ToM are subserved by distinct, yet interacting neural structures (Baron-Cohen 1995; Sabbagh 2004). The specific roles of the different regions are still debated, however, with some studies arguing that the MPFC plays a key role in mental state reasoning (Gallagher and Frith 2003; Sabbagh 2004) and others arguing that the TPJ is the key player (Saxe et al. 2009; Gweon et al. 2012). Given that the anterior third of the CC connects the prefrontal cortices, the former studies align with our finding that larger mid-anterior (genu) and central (anterior mid-body) volumes correlate with better ToM abilities. These results are also in concordance with a study by Kana et al. (2009), which found that, in adults with ASD, the frontal lobe is the “epicenter of underconnectivity” during mental state reasoning. It may thus be hypothesized that a larger mid-anterior and central CC increase the capacity for interhemispheric communication between the prefrontal cortices, allowing for better performance on ToM tasks.

Moreover, it is important to note that, even though adult neuroimaging studies have identified more posterior brain regions involved in ToM, including the TPJ and the temporal poles (Frith and Frith 2007; Burnett and Blakemore 2009), child neuroimaging studies have been a lot less consistent. While some studies have also found the bilateral TPJ to play an important role during ToM tasks (Saxe et al. 2009; Gweon et al. 2012; Mukerji et al. 2019), other studies have shown only the MPFC to be reliably active in children (Kobayashi et al. 2007b; Liu et al. 2009). It has been proposed that the MPFC is more important for the universal understanding of ToM during childhood than during adulthood, and that the TPJ becomes increasingly selective for mental state reasoning during development (Saxe et al. 2009; Gweon et al. 2012). Additionally, adolescents show decreasing prefrontal activity with age during ToM tasks, suggesting an anterior-to-posterior shift in cortical activity from preadolescence into adulthood (Blakemore 2012). This hypothesis may thus further explain our results, which only showed the volumes of the anterior portion of the CC to be significantly correlated with ToM in school-aged children.

### Age-related differences in the relationship between CC subsection volumes and ToM abilities

The finding that the older age group drives the association between CC mid-anterior and central subsection volumes and ToM abilities also complements the hypothesis of an anterior-to-posterior maturation gradient (Thompson et al. 2000). According to this hypothesis, the most pronounced growth in anterior sections of the CC occurs in early childhood, around 3–6 years of age. Growth in posterior sections then begins to dominate, with one study reporting ages 9–12 to be a critical period for this switch (Luders et al. 2010). In the present study, this would suggest that 9- to 12-year-old children whose anterior CC was still growing performed worse on ToM tasks than children where the anterior-to-posterior switch had already occurred. In younger children, where the anterior CC is still developing, subsection volume may not be a predictor of ToM abilities yet, which would explain the non-significant results in the 6- to 8-year-olds in this study. In other words, subsection volumes may only become a predictor of ToM abilities later in development.

Regarding the ToM subscales, we found that larger mid-anterior and central subsection volumes correlated with better ToM 2 and ToM 3 abilities in the older group only. Given that ToM 1 assesses precursors of ToM, including emotion recognition and distinction between reality and non-reality, children aged 9–12 years generally do well on this subscale. This ceiling effect is reflected by our results, which do not show an association between the CC volume and ToM 1. The subsequent subscales, however, assess children’s understanding of first- and second-order false beliefs, which are more challenging than emotion recognition and require multiple steps of processing. Therefore, the influence of CC volume may only become apparent at higher levels of ToM and at an age where the anterior sections should have largely developed.

### Sex-related differences in the relationship between CC subsection volumes and ToM abilities

Our third finding relates to the sex differences in the correlation between certain CC subsection volumes and ToM abilities. To start, we did not find sex differences in ToM abilities in our sample, which aligns with previous studies demonstrating that boys and girls are comparable in this domain (Holmes et al. 1996; Jenkins and Astington 1996; Charman et al. 2002). However, there is some evidence that girls perform better on ToM-related tasks, including mental state understanding (Hughes and Dunn 1998), emotion recognition (Dunn et al. 1991), and empathy (Baron-Cohen et al. 1999), especially in older children and adolescents. Studies in younger children do not seem to find this female advantage (Hughes et al. 2011), which may explain the lack of sex differences in ToM abilities seen in the present sample of 6- to 12-year-old children.

Our study did, however, reveal significant sex differences in the relationship between CC volume and ToM abilities. More specifically, we found a larger mid-anterior subsection volume to correlate with better overall ToM abilities in girls and a larger central subsection volume to correlate with better overall ToM abilities in boys. One possible explanation for these differences may be that girls and boys use different strategies to solve ToM tasks. These strategic differences could lead to different activation patterns in the cortex, which would in turn be reflected in specific subsections of the CC that correspond to the cortical region from where those fibers originate (Newman 2016). Therefore, sex differences as a function of ToM may not be seen in the entire CC, but only in the subsections connecting cortical areas related to ToM, which may differ between males and females. In fact, several studies in adolescents and adults have shown that females activate frontal regions and the superior temporal sulcus more, while males activate the TPJ and precuneus more during various socio-cognitive tasks (Schulte-Ruther et al. 2008; Veroude et al. 2014; Adenzato et al. 2017; Gao et al. 2019). However, since the central subsection does not connect the regions that are more active during ToM in males, the present sex differences may also indicate that CC volume is a better predictor of ToM abilities in females than males.

Interestingly, we also found a smaller anterior volume to correlate with better performance on precursors of ToM, such as emotion recognition, in boys only. According to the theory of excitation, a smaller CC indicates greater functional asymmetry (Bloom and Hynd 2005). In the context of the present study, this would suggest that the degree of lateralization and the cortical activation patterns during emotion recognition (i.e. ToM 1) differ between males and females. While previous studies examining sex-related differences in emotion recognition found greater limbic, inferior frontal, and temporal cortex activation in females and greater prefrontal and parietal activation in males, it remains contested whether there are also differences in the degree of lateralization during emotion recognition (see Whittle et al. 2011 for a review). However, one study in adults found greater laterality towards the right hemisphere during facial emotion perception among males and a more diffuse lateralization among females (Harrison et al. 1990). It may thus be posited that male children who already display greater functional asymmetry during tasks measuring precursors of ToM perform better than male children who recruit cortical areas bilaterally. In females, on the other hand, functional asymmetry may not be an indicator of performance on basic emotion recognition tasks.

### Limitations and future directions

The present study also has some limitations that should be addressed. First, our sample size was relatively small, especially when we split the sample into age- and sex-based groups. The age- and sex-related findings should therefore be considered preliminary and interpreted with caution until replicated in larger samples. However, given the lack of data on the relationship between CC volume and ToM abilities in healthy children, this study provides important first insights into the potential role that the structure of the CC may play in the typical development of ToM. Future studies should also use functional connectivity data to elucidate the relationship between the CC and ToM abilities in typically developing populations. Moreover, the interpretability of our data was limited by the lack of normative data available for the ToM subscales. While we controlled for age in the analyses involving ToM subscales, we are aware of a possible non-linear relationship between age and ToM; hence, analyses involving ToM subscales should also be interpreted with caution. Finally, it is possible that a third variable explains the presently found associations, such as a broader deficit in information integration or processing speed. Studies have shown that processing speed is strongly linked to performance on standardized tests, executive functioning, and problem solving (Fischer et al. 1992; Brown and Paul 2000). In turn, it has been posited that deficits in these domains can result in impaired social skills, including ToM (Symington et al. 2010; Turk et al. 2010). Since the present study did not assess the children’s processing speed, we were unable to control for this potential confounder. Future studies should thus consider also measuring participants’ processing speed and problem solving abilities when examining their performance on ToM tasks.

## Conclusions

This study is the first to show that the volumes of the mid-anterior and central CC subsections are associated with the ToM abilities of typically developing children. The children’s age further influenced this relationship, with the 9- to 12-year-olds driving the association. We interpreted this age effect in light of the anterior-to-posterior maturation hypothesis, which posits ages 9–12 to be the period during which growth in posterior sections begins to dominate over growth in anterior sections. Together, these findings elucidate which subsections of the CC are involved in supporting social cognition during various developmental periods. The study also addresses an important gap in the current literature, as it provides evidence that CC volume is related to variation in healthy children’s ToM abilities. CC subsection volumes may thus serve as a valuable metric of heterogeneity both in typical development and in neurodevelopmental populations known to exhibit socio-cognitive deficits.

## Supplementary Material

Supplement_bhad353

## Data Availability

The datasets generated and analyzed during the present study are not publicly available due to privacy and ethical restrictions but are available in anonymized form upon reasonable request.
