# Supplementary material for: Larger corpus callosum volume is favorable for theory of mind development in healthy children
Source: Cereb Cortex. 2023 Oct 11;33(23):11197–205. doi: 10.1093/cercor/bhad353 (PMC10690855; doi:10.1093/cercor/bhad353)
Supplement: Supplement_bhad353 [file supplement_bhad353.docx]

**Supplementary Information**

**Table S1.** Participants’ performance on the cognitive tests and the theory of mind task based on sex and age.

|  | Total  (N) | Sex  (female/male) | Age range  (years) |
| --- | --- | --- | --- |
| Language comprehension |  |  |  |
| Average | 23 | 11/12 | 6.33-12.25 |
| Above Average | 13 | 7/6 | 7.17-12.67 |
| Below Average | 1 | 0/1 | 8.83 |
| Impaired | - | - | - |
| Expressive vocabulary |  |  |  |
| Average | 19 | 12/7 | 6.33-12.25 |
| Above Average | 9 | 3/6 | 7.50-12.67 |
| Below Average | 6 | 2/4 | 6.42-12.08 |
| Impaired | 3 | 1/2 | 8.83-10.75 |
| Perceptual reasoning |  |  |  |
| Average | 25 | 14/11 | 6.42-12.67 |
| Above Average | 8 | 3/5 | 7.50-10.08 |
| Below Average | 3 | 2/1 | 6.33-12.25 |
| Impaired | 1 | 0/1 | 8.83 |
| Theory of mind |  |  |  |
| Average | 14 | 7/7 | 6.33-12.67 |
| Above Average | 8 | 5/3 | 6.50-10.33 |
| Below Average | 10 | 3/7 | 7.08-12.25 |
| Impaired | 5 | 3/2 | 7.50-11.67 |

|  | Lang. comprehension *r* (p) | Expressive vocabulary *r* (p) | Perceptual reasoning  *r* (p) |
| --- | --- | --- | --- |
| CC total | -.12 (.475) | .03 (.877) | .02 (.895) |
| CC anterior | .08 (.624) | .05 (.787) | -.00 (.987) |
| CC mid-anterior | -.23 (.173) | -.26 (.114) | .05 (.766) |
| CC central | -.10 (.544) | -.06 (.722) | .11 (.521) |
| CC mid-posterior | -.01 (.946) | .09 (.581) | .01 (.976) |
| CC posterior | -.17 (.303) | .19 (.257) | -.07 (.672) |

**Table S2.** Correlations between corpus callosum volumes and language comprehension, expressive vocabulary, and perceptual reasoning.

Note: CC = corpus callosum.

**Table S3**. Correlations among corpus callosum volumes, ToM subtest raw scores, and ToM total z-scores in the whole group.

|  | ToM 1 raw  *r* (p) | ToM 2 raw  *r* (p) | ToM 3 raw  *r* (p) | ToM total z-score  *r* (p) |
| --- | --- | --- | --- | --- |
| CC total | .03 (.867) | .09 (.612) | .25 (.142) | .26 (.119) |
| CC anterior | -.26 (.132) | -.09 (.589) | .01 (.934) | -.04 (.799) |
| CC mid-anterior | .18 (.297) | .03 (.846) | .29 (.083) | **.36 (.030)*** |
| CC central | **.34 (.043)*** | .17 (.336) | **.34 (.030)*** | **.46 (.005)*** |
| CC mid-posterior | .19 (.270) | .14 (.402) | .15 (.369) | .16 (.334) |
| CC posterior | -.22 (.198) | .07 (.673) | .08 (.636) | .04 (.805) |

Note: CC = corpus callosum, ToM = theory of mind. ToM 1-3 were controlled for age using partial correlation analyses, whereas Pearson’s correlation was used for the ToM total z-scores. Significant correlations are indicated in bold and marked with an asterisk (*).

**Table S4**. Correlations among corpus callosum volumes, ToM subtest raw scores, and ToM total z-scores in 9- to 12-year-olds.

|  | ToM 1 raw  *r* (p) | ToM 2 raw  *r* (p) | ToM 3 raw  *r* (p) | ToM total z-score  *r* (p) |
| --- | --- | --- | --- | --- |
| CC total | .02 (.948) | **.51 (.038)*** | .42 (.091) | .38 (.124) |
| CC anterior | -.25 (.335) | .29 (.262) | .12 (.638) | .03 (.915) |
| CC mid-anterior | .16 (.546) | **.63 (.007)*** | **.63 (.007)*** | **.61 (.007)*** |
| CC central | .37 (.140) | .46 (.063) | **.52 (.033)*** | **.58 (.012)*** |
| CC mid-posterior | .07 (.790) | .17 (.522) | .20 (.431) | .18 (.472) |
| CC posterior | -.21 (.420) | .34 (.187) | .15 (.559) | .09 (.735) |

Note: CC = corpus callosum, ToM = theory of mind. ToM 1-3 were controlled for age using partial correlation analyses, whereas Pearson’s correlation was used for the ToM total z-scores. Significant correlations are indicated in bold and marked with an asterisk (*).
